# Supplementary material for: Comparing antiviral strategies against COVID-19 via multiscale within-host modelling
Source: R Soc Open Sci. 2021 Aug 11;8(8):210082. doi: 10.1098/rsos.210082 (PMC8355669; doi:10.1098/rsos.210082)
Supplement: Supplementary file 1 [file rsos210082_review_history.pdf]

THE ROYAL SOCIETY
PUBLISHING

ROYAL SOCIETY
OPEN SCIENCE

Comparing antiviral strategies against COVID-19 via multiscale within-host modelling

F. Fatehi, R. J. Bingham, E. C. Dykeman, P. G. Stockley and R. Twarock

Article citation details
R. Soc. open sci. **8**: 210082.
http://dx.doi.org/10.1098/rsos.210082

Review timeline

Original submission:	21 January 2021
Revised submission:	30 June 2021
Final acceptance:	23 July 2021

Note: Reports are unedited and appear as submitted by the referee. The review history appears in chronological order.

Note: This manuscript was transferred from another Royal Society journal with peer review.

Review History

RSOS-210082.R0 (Original submission)

Review form: Reviewer 1

Is the manuscript scientifically sound in its present form?
Yes

Are the interpretations and conclusions justified by the results?
Yes

Is the language acceptable?
Yes

Do you have any ethical concerns with this paper?
No

Have you any concerns about statistical analyses in this paper?
No

Reports © 2021 The Reviewers; Decision Letters © 2021 The Reviewers and Editors; Responses © 2021 The Reviewers, Editors and Authors. Published by the Royal Society under the terms of the Creative Commons Attribution License http://creativecommons.org/licenses/by/4.0/, which permits unrestricted use, provided the original author and source are credited

Recommendation?
Accept with minor revision (please list in comments)

Comments to the Author(s)
The authors successfully answered a number of my questions. However, I am still unconvinced about some aspects of the statistical analysis and model fitting. I believe these question require a new round of review:
1/ You argue that estimation with Monolix was not possible thus you fitted the model using individual by individual approach in Matlab. First, to avoid any confusion, I believe that the references to Monolix should be removed to avoid confusions - just present the methods you used in more details. Second, can you properly check the identifiability of your model (for exemple the method of Castro and de boer 2020 could be used). I am concerned by estimates stability and over-interpretation. It may be that some parameters compensate each other and it would be more suitable to fix some parameters to some values in order to achieve identifiability.
2/ Even if you do individual by individual fittings, it is perfectly possible to get confidence intervals. This is possible doing bootstrap using Monte Carlo simulations. You can samplevalues on the multivariate posterior distribution of parameters and sample possible trajectories. Then just take the 2.5% and 97.5% percentile of the sampled trajectories. See Appendix A.

Decision letter (RSOS-210082.R0)

We hope you are keeping well at this difficult and unusual time. We continue to value your support of the journal in these challenging circumstances. If Royal Society Open Science can assist you at all, please don't hesitate to let us know at the email address below.

Dear Dr Twarock

On behalf of the Editors, we are pleased to inform you that your Manuscript RSOS-210082 "Comparing antiviral strategies against COVID-19 via multiscale within-host modelling" has been accepted for publication in Royal Society Open Science subject to minor revision in accordance with the referees' reports. Please find the referees' comments along with any feedback from the Editors below my signature.

We invite you to respond to the comments and revise your manuscript. Below the referees' and Editors' comments (where applicable) we provide additional requirements. Final acceptance of your manuscript is dependent on these requirements being met. We provide guidance below to help you prepare your revision.

Please submit your revised manuscript and required files (see below) no later than 7 days from today's (ie 17-May-2021) date. Note: the ScholarOne system will 'lock' if submission of the revision is attempted 7 or more days after the deadline. If you do not think you will be able to meet this deadline please contact the editorial office immediately.

Please note article processing charges apply to papers accepted for publication in Royal Society Open Science (https://royalsocietypublishing.org/rsos/charges). Charges will also apply to papers transferred to the journal from other Royal Society Publishing journals, as well as papers submitted as part of our collaboration with the Royal Society of Chemistry (https://royalsocietypublishing.org/rsos/chemistry). Fee waivers are available but must be requested when you submit your revision (https://royalsocietypublishing.org/rsos/waivers).

Thank you for submitting your manuscript to Royal Society Open Science and we look forward to receiving your revision. If you have any questions at all, please do not hesitate to get in touch.

Kind regards,
Royal Society Open Science Editorial Office
Royal Society Open Science
openscience@royalsociety.org

on behalf of Professor Tim Rogers (Associate Editor) and Mark Chaplain (Subject Editor)
openscience@royalsociety.org

Associate Editor Comments to Author (Professor Tim Rogers):
Associate Editor: 1
Comments to the Author:
Given the high importance of the topic, we must apply a high standard for the statistical analyses - please address the concerns of the referee in full.

Reviewer comments to Author:
Reviewer: 1

Comments to the Author(s)
The authors successfully answered a number of my questions. However, I am still unconvinced about some aspects of the statistical analysis and model fitting. I believe these question require a new round of review:
1/ You argue that estimation with Monolix was not possible thus you fitted the model using individual by individual approach in Matlab. First, to avoid any confusion, I believe that the references to Monolix should be removed to avoid confusions - just present the methods you used in more details. Second, can you properly check the identifiability of your model (for exemple the method of Castro and de boer 2020 could be used). I am concerned by estimates stability and over-interpretation. It may be that some parameters compensate each other and it would be more suitable to fix some parameters to some values in order to achieve identifiability.
2/ Even if you do individual by individual fittings, it is perfectly possible to get confidence intervals. This is possible doing bootstrap using Monte Carlo simulations. You can samplevalues on the multivariate posterior distribution of parameters and sample possible trajectories. Then just take the 2.5% and 97.5% percentile of the sampled trajectories.

===PREPARING YOUR MANUSCRIPT===

Your revised paper should include the changes requested by the referees and Editors of your manuscript. You should provide two versions of this manuscript and both versions must be provided in an editable format:
one version identifying all the changes that have been made (for instance, in coloured highlight, in bold text, or tracked changes);
a 'clean' version of the new manuscript that incorporates the changes made, but does not highlight them. This version will be used for typesetting.

Please ensure that any equations included in the paper are editable text and not embedded images.

Please ensure that you include an acknowledgements' section before your reference list/bibliography. This should acknowledge anyone who assisted with your work, but does not qualify as an author per the guidelines at https://royalsociety.org/journals/ethics-policies/openness/.

While not essential, it will speed up the preparation of your manuscript proof if you format your references/bibliography in Vancouver style (please see https://royalsociety.org/journals/authors/author-guidelines/#formatting). You should include DOIs for as many of the references as possible.

If you have been asked to revise the written English in your submission as a condition of publication, you must do so, and you are expected to provide evidence that you have received language editing support. The journal would prefer that you use a professional language editing service and provide a certificate of editing, but a signed letter from a colleague who is a native speaker of English is acceptable. Note the journal has arranged a number of discounts for authors using professional language editing services (https://royalsociety.org/journals/authors/benefits/language-editing/).

===PREPARING YOUR REVISION IN SCHOLARONE===

To revise your manuscript, log into https://mc.manuscriptcentral.com/rsos and enter your Author Centre - this may be accessed by clicking on "Author" in the dark toolbar at the top of the page (just below the journal name). You will find your manuscript listed under "Manuscripts with Decisions". Under "Actions", click on "Create a Revision".

Attach your point-by-point response to referees and Editors at Step 1 'View and respond to decision letter'. This document should be uploaded in an editable file type (.doc or .docx are preferred). This is essential.

Please ensure that you include a summary of your paper at Step 2 'Type, Title, & Abstract'. This should be no more than 100 words to explain to a non-scientific audience the key findings of your research. This will be included in a weekly highlights email circulated by the Royal Society press office to national UK, international, and scientific news outlets to promote your work.

At Step 3 'File upload' you should include the following files:
-- Your revised manuscript in editable file format (.doc, .docx, or .tex preferred). You should upload two versions:
1) One version identifying all the changes that have been made (for instance, in coloured highlight, in bold text, or tracked changes);
2) A 'clean' version of the new manuscript that incorporates the changes made, but does not highlight them.
-- An individual file of each figure (EPS or print-quality PDF preferred [either format should be produced directly from original creation package], or original software format).
-- An editable file of each table (.doc, .docx, .xls, .xlsx, or .csv).
-- An editable file of all figure and table captions.
Note: you may upload the figure, table, and caption files in a single Zip folder.
-- Any electronic supplementary material (ESM).
-- If you are requesting a discretionary waiver for the article processing charge, the waiver form must be included at this step.
-- If you are providing image files for potential cover images, please upload these at this step, and inform the editorial office you have done so. You must hold the copyright to any image provided.
-- A copy of your point-by-point response to referees and Editors. This will expedite the preparation of your proof.

At Step 6 'Details & comments', you should review and respond to the queries on the electronic submission form. In particular, we would ask that you do the following:

-- Ensure that your data access statement meets the requirements at https://royalsociety.org/journals/authors/author-guidelines/#data. You should ensure that you cite the dataset in your reference list. If you have deposited data etc in the Dryad repository, please only include the 'For publication' link at this stage. You should remove the 'For review' link.
-- If you are requesting an article processing charge waiver, you must select the relevant waiver option (if requesting a discretionary waiver, the form should have been uploaded at Step 3 'File upload' above).
-- If you have uploaded ESM files, please ensure you follow the guidance at https://royalsociety.org/journals/authors/author-guidelines/#supplementary-material to include a suitable title and informative caption. An example of appropriate titling and captioning may be found at https://figshare.com/articles/Table_S2_from_Is_there_a_trade-off_between_peak_performance_and_performance_breadth_across_temperatures_for_aerobic_scope_in_teleost_fishes_/3843624.

At Step 7 'Review & submit', you must view the PDF proof of the manuscript before you will be able to submit the revision. Note: if any parts of the electronic submission form have not been completed, these will be noted by red message boxes.

Author's Response to Decision Letter for (RSOS-210082.R0)

See Appendix B.

Decision letter (RSOS-210082.R1)

We hope you are keeping well at this difficult and unusual time. We continue to value your support of the journal in these challenging circumstances. If Royal Society Open Science can assist you at all, please don't hesitate to let us know at the email address below.

Dear Dr Twarock,

It is a pleasure to accept your manuscript entitled "Comparing antiviral strategies against COVID-19 via multiscale within-host modelling" in its current form for publication in Royal Society Open Science.

COVID-19 rapid publication process:
We are taking steps to expedite the publication of research relevant to the pandemic. If you wish, you can opt to have your paper published as soon as it is ready, rather than waiting for it to be published the scheduled Wednesday.

This means your paper will not be included in the weekly media round-up which the Society sends to journalists ahead of publication. However, it will still appear in the COVID-19 Publishing Collection which journalists will be directed to each week (https://royalsocietypublishing.org/topic/special-collections/novel-coronavirus-outbreak).

If you wish to have your paper considered for immediate publication, or to discuss further, please notify openscience_proofs@royalsociety.org and press@royalsociety.org when you respond to this email.

You can expect to receive a proof of your article in the near future. Please contact the editorial office (openscience@royalsociety.org) and the production office (openscience_proofs@royalsociety.org) to let us know if you are likely to be away from e-mail contact -- if you are going to be away, please nominate a co-author (if available) to manage the proofing process, and ensure they are copied into your email to the journal.

Due to rapid publication and an extremely tight schedule, if comments are not received, your paper may experience a delay in publication.

Please see the Royal Society Publishing guidance on how you may share your accepted author manuscript at https://royalsociety.org/journals/ethics-policies/media-embargo/. After publication, some additional ways to effectively promote your article can also be found here https://royalsociety.org/blog/2020/07/promoting-your-latest-paper-and-tracking-your-results/.

Thank you for your fine contribution. On behalf of the Editors of Royal Society Open Science, we look forward to your continued contributions to the Journal.

Kind regards,
Royal Society Open Science Editorial Office
Royal Society Open Science
openscience@royalsociety.org

on behalf of Professor Tim Rogers (Associate Editor) and Mark Chaplain (Subject Editor)
openscience@royalsociety.org

Follow Royal Society Publishing on Twitter: @RSocPublishing
Follow Royal Society Publishing on Facebook:
https://www.facebook.com/RoyalSocietyPublishing.FanPage/
Read Royal Society Publishing's blog:
https://royalsociety.org/blog/blogsearchpage/?category=Publishing

Appendix A

Review – Fatehi et al. - Comparing antiviral strategies against 1 COVID-19 via multi-scale within host 2 modelling

The article uses a stochastic agent-based models and ODE-based dynamical models to help understanding the within-host infection dynamics of COVID-19. They investigate in silico the effect of remdesivir and convalescent plasma therapy and their possible synergetic interaction.

The supplemental information file was not available (it was a duplicate of the main manuscript). It made it difficult to understand the details of simulations and results. This file will need to be provided for further review.

The author calibrated their model on 12 patients from a study in Singapore (Young et al.) using the Monolix software. They also based their model on available experimental data. For some parameters, they investigate the robustness of their conclusion to variation in the parameters value using sensibility analysis. Although the approach is valid, I have remaining question regarding the identifiability of the model and the robustness of their estimation. In particular, more confidence interval should be provided to appreciate the quality of the results.

Their primary endpoints to compare scenario for the study is the viral load that is secreted from infected cells and tissue damage. The later notion is not very well defined in the manuscript. Trade-off between the two aspects are also not very much discussed. Finally, discussion can be expended to discuss more clinical implication in particular on symptoms and disease severity.

The results provided on the effect of convalescent plasma and antiviral therapies are expected. In particular, the fact that these therapies are most effective if administered early post infection. However, the modeling work in itself provide quantification and represent a good platform for further experiments. In silico simulation of the counterfactual effect of therapies in the 12 patients from the Singapore study could be further developed (although partially developed).

In general, article will benefit from some writing efforts. The modeling and the findings are interesting, but their impact is decreased by the number of assumptions made in the modeling. The effort made by the authors to make these assumptions clear is good, but they are given here and there in the text which impacts the clarity. Organization of the paper should be revised so that modeling is presented all at once (especially for the CP and antiviral therapies effects) with its assumptions. To do so, material and method section could be expended.

Major comments

1. More references need to be added in the introduction. First there is no string review of existing models of immune response to viral infection. Second, there is a growing existing literature on clinical results of remdesivir and plasma convalescent therapy effect. This should be reviewed to expose in the introduction the scientific and medical interest of this in silico research.

2. Section 2.1/2.2, there is a gap between the modeling presented and the results. Please explain what is done more explicitly in the main manuscript which should stand alone without the appendix. Part of the material of this section could be described in Material and methods.
3. In material and method, you mention *"However, considering a higher basal level does not change model outcome as a and μ which determine the proliferation rate of effector cells and removal rate of infected cells by effector cells are estimated using data fitting."* This is not clear to me at all. Do you refer to non-identifiability? Did you perform an identifiability analysis for you model? Same question also applies for the estimation of other parameters.
4. You use article in HBV to set parameters values in your model, for example for antibodies death rate (as well as other parameters). Can you discuss how realistic this choice is?
5. When using Monolix did you fit each patients' data separately? If yes, this is known to be suboptimal compared to a population fitting using mixed effects, which I believe you did. Please clarify.
6. Regarding the use of Monolix and table 1, can you provide the standard deviation for parameter estimation. This indicator is different from the std you reported in the table. You get them by computing the Fisher information matrix. On top of providing information about the model identifiability, this would provide insight on uncertainty in parameters estimation. Can you also provide in appendix an idea of the fit to the data (visual predictive check and/or individual data fits)? The analysis would also benefit from a thorough convergence assessment procedure as proposed by Monolix.
7. Is the multi-peak behavior of the viral load something reported in clinical data /other studies? Could it be some well-known overshooting/oscillatory behavior only due to the mechanics of ODE systems?
8. The assumption of Remdesivir efficacy is very strong. 0.99 seems to be very high compared to values found in the literature (Gonçalves, A., Bertrand, J., Ke, R., Comets, E., De Lamballerie, X., Malvy, D., ... & Smith, P. (2020). Timing of antiviral treatment initiation is critical to reduce SARS-CoV-2 viral load. *CPT: pharmacometrics & systems pharmacology*, *9*(9), 509-514).
9. In Figure 3, the discussion of the number of days before starting the treatment is not convincing. Why the dotted line (dpi=6 days) does not appear in most graphics? AUC is advocated as a good indicator. Can the author provide the values of AUC as well as confidence interval for them? For example, l281 p10/16, you mention that AUC is reduced significantly. Please provide quantification of it as well as for the significance.
10. Confidence intervals are never provided making it difficult to appreciate the uncertainty in the results. Please add them, including in graphics.
11. Individual prediction. Simulation in silico of CP and antiviral therapy in the 12 patients of the Singapore study is interesting. Please provide more details in the main manuscript.
12. The severity of symptoms is known to be associated with a higher antibodies level in patients. How does this information relate with your model? Please at least mention some discussion points in the discussion part.

Minor comments

1. The introduction should be revised so that the results of the article are not presented in end of the introduction.
2. l118 p3/16, Please provide references for “experimentally available data”.
3. Figures need to be readable in black and white. Please edit them. I believe this should be possible for all of them.
4. l129 p4/16, refer to the figures by their numbers.
5. Make the conversion between Ct and viral copies more explicit.
6. Some acronyms are not spelled out.
7. Describe the shape of the “Heavyside” function.
8. In the abstract you only mention ABM model. However more than half of the paper is based on ODE models with mixed effects on parameters. Please revise.

Appendix B

We are very grateful to the Reviewer for their careful reading of our manuscript and their helpful comments. We address their questions, point-by-point below.

1/ The authors successfully answered a number of my questions. However, I am still unconvinced about some aspects of the statistical analysis and model fitting. I believe these question require a new round of review:

You argue that estimation with Monolix was not possible thus you fitted the model using individual by individual approach in Matlab. First, to avoid any confusion, I believe that the references to Monolix should be removed to avoid confusions - just present the methods you used in more details. Second, can you properly check the identifiability of your model (for exemple the method of Castro and de boer 2020 could be used). I am concerned by estimates stability and over-interpretation. It may be that some parameters compensate each other and it would be more suitable to fix some parameters to some values in order to achieve identifiability.

We would like to thank the reviewer for suggesting the Castro and de Boer method, that we used to establish the structural identifiability of our model (lines 464-465). We removed the reference to Monolix and added more details about the method used here (lines 467-568).

2/ Even if you do individual by individual fittings, it is perfectly possible to get confidence intervals. This is possible doing bootstrap using Monte Carlo simulations. You can samplevalues on the multivariate posterior distribution of parameters and sample possible trajectories. Then just take the 2.5% and 97.5% percentile of the sampled trajectories.

We used the method suggested by the reviewer to compute bootstrap 95% CIs for the parameter estimates in Table S2 (lines 474-476). We also included 95% CI into the patient data fitting figure (Fig. S4) and updated its caption. However, because of the negligible width of the curves, these shaded areas are not visible. We plotted the same figure without the fitted curves to illustrate that the 95% CI shaded areas are indeed very small (see figure below).

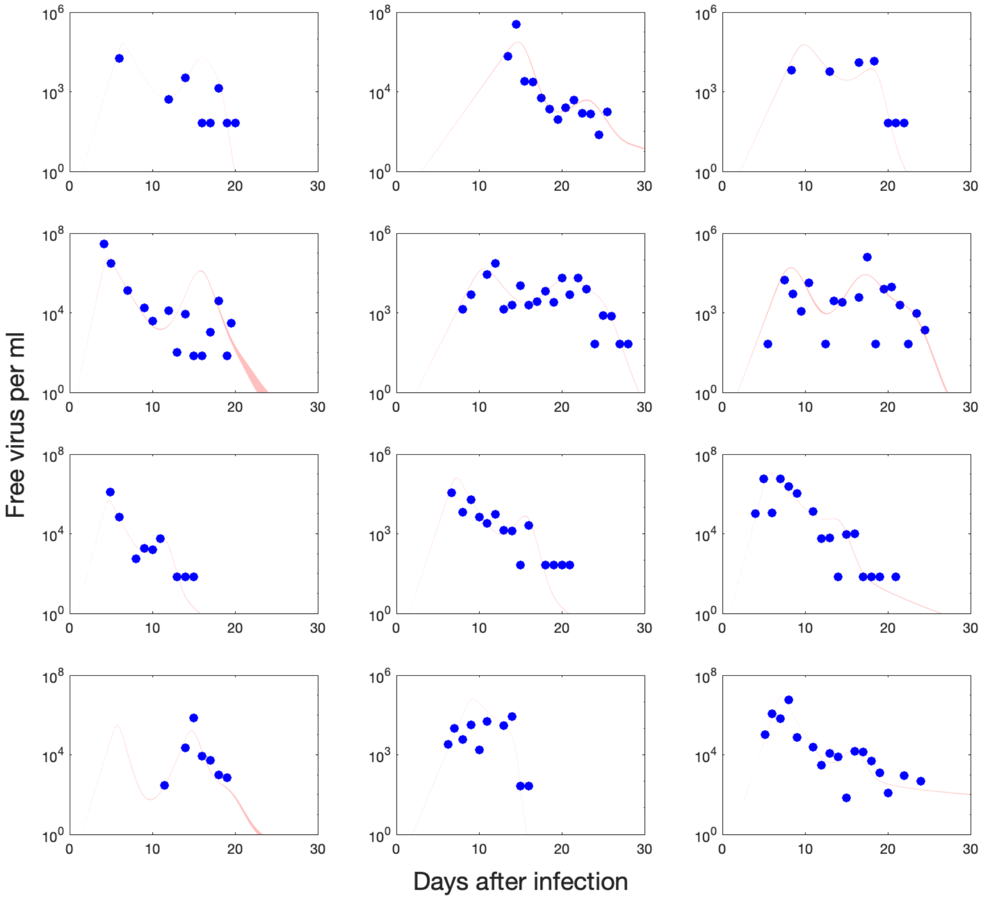
Free virus per ml
Days after infection
